# Enhanced understanding of nitrogen fixing bacteria through DNA extraction with polyvinylidene fluoride membrane

**DOI:** 10.1038/s41598-025-00173-5

**Published:** 2025-05-08

**Authors:** Agnieszka Kalwasińska, Igor Królikiewicz, Sushma Rani Tirkey, Attila Szabó, Sweta Binod Kumar

**Affiliations:** 1 Department of Environmental Microbiology and Biotechnology, Faculty of Biological and Veterinary Sciences, Nicolaus Copernicus University in Toruń, Lwowska 1, Toruń, 87-100 Poland; 2 Department of Aquatic Sciences and Assessment, Swedish University of Agricultural Sciences, Uppsala, 750 07 Sweden; 3 Institute of Aquatic Ecology, HUN-REN Centre for Ecological Research, Karolina út 29, Budapest, 1113 Hungary

**Keywords:** Bacterial diversity, Nitrogen-fixing bacteria, *nif*H, PVDF membrane, Rhizosphere, Ecology, Metagenomics

## Abstract

The rhizobiota, particularly nitrogen-fixing bacteria, play a crucial role in plant functioning by providing essential nutrients and defense against pathogens. This study investigated the diversity of nitrogen-fixing bacteria in a relatively understudied habitat: technosoils developed from industrial soda production. To analyze the bacterial diversity in the rhizosphere soils of wheat (*Triticum aestivum* L.) and aster (*Tripolium pannonicum* Jacq.), regions of the *nif*H gene were amplified and sequenced from the resident bacterial communities. A polyvinylidene fluoride (PVDF) membrane was employed for metagenomic DNA extraction, enhancing the detection of nitrogen-fixing bacteria. Prior to standard DNA extraction, an enrichment step was conducted in nitrogen-free JMV medium at 26 °C for 24 h, with a modification that replaced soil with the PVDF membrane. This approach enabled a more comprehensive analysis of the rhizosphere bacterial community, revealing that unique amplicon sequence variants (ASVs) in aster and wheat membrane samples accounted for a notable proportion of all ASVs in the dataset (8.5% and 23%, respectively) that were not captured using the standard method. Additionally, our findings demonstrated higher alpha diversity of nitrogen-fixing bacteria in the wheat rhizosphere compared to the aster rhizosphere. In wheat, the dominant genus was *Insolitispirillum* (38.80%), followed by unclassified genera within Gammaproteobacteria (9.76%) and *Rhodospirillaceae* (4.74%). In contrast, the aster rhizosphere was predominantly occupied by *Azotobacter* (95.69%).

**Supplementary Information:**

The online version contains supplementary material available at 10.1038/s41598-025-00173-5.

## Introduction

Polyvinylidene fluoride (PVDF) is a hydrophobic, thermally stable material that possesses great mechanical strength. It is resistant to chemical corrosion, oxidation, and pollution and is low-cost to produce. These qualities make it a popular membrane material in various industrial fields^1,2^. PVDF membranes are integral parts of seawater desalination, and wastewater microfiltration processes^3,4^. However, biofilm formation contributes to the blocking of the pores, reduction of filtration efficiency, shortens the lifespan of the membrane and increases energy consumption in membrane technologies in wastewater treatment applications. It has been reported that the PVDF membrane possesses a high affinity towards various bacteria due to its high hydrophobicity, roughness, and the accumulation of trace organic nutrients at the solid-liquid interface^5,6^.

The adhesion of bacteria to the membrane results from a combination of bacterial and material characteristics. Bacteria, like other microorganisms, have a charged cell wall due to acid-based groups on their surface. Their cell walls are mostly hydrophobic, a crucial factor in strong adherence to the material’s hydrophobic surfaces^7^. However, hydrophobicity isn’t the sole factor influencing adhesion. Surface charge, roughness, chemical composition, pH, temperature, and ionic strength of the membrane surface and surrounding environment can significantly impact bacterial attachment^8^.

The results of research by Kumar et al.^5^ indicate that the bacterial community recovered from the PVDF membrane dipped in sample water was more diverse compared to the community obtained directly from the same water samples. This discovery served as the foundation for developing distinct sampling tools i.e. membrane-based kits in the form of strips for the detection of *E. coli*^9^ and *Vibrio* species (*V. cholerae*,* V. parahaemolyticus*,* V. campbellii*,* V. harveyi*, and *V. proteolyticus*) in water samples^10^.

In this study, we aimed to evaluate the effectiveness of PVDF membranes in the pre-treatment step to enhance bacterial detection in soil environments. DNA extraction from soil generally involves isolating genetic material from microorganisms within the soil matrix using a combination of physical, chemical, and enzymatic methods. Common approaches include direct methods, which lyse soil cells to maximize yield, and indirect methods, which separate cells before lysis to minimize contaminants, such as humic acids, that can interfere with downstream analyses^11^. The use of a PVDF membrane, which concentrates bacteria and allows them to proliferate before conventional DNA extraction, may improve the detection of rare taxa present in small quantities within the soil. To assess bacterial diversity, amplicon sequencing of the *nif*H gene on the Illumina platform was employed. Our focus was specifically on nitrogen-fixing bacteria from the rhizosphere, which have been extensively studied for their unique ability to fix atmospheric nitrogen and convert it into a plant-available form, offering potential to reduce agriculture’s chemical dependence^12–14^.

Recent microbiological research has been extensively conducted in northern Poland to investigate how environmental parameters influence microbial abundance, structure, diversity, and, in particular, specific guilds involved in the nitrogen cycle in saline soils affected by the soda industry^15^. Our findings indicated that salinity had less impact on microorganisms responsible for nitrogen fixation and denitrification compared to the nitrifying guild. Notably, the diversity of nitrogen-fixing communities had not been previously explored in this region. Therefore, a secondary goal of our study was to uncover the diversity of nitrogen-fixing bacteria in technosoils from Inowrocław. The research hypotheses were as follows: (i) Soil DNA extraction using a PVDF membrane during the enrichment step is more efficient, resulting in higher bacterial diversity compared to the standard method without enrichment. (ii) Nitrogen-fixing bacteria in soda-impacted technosoils form a unique community when compared to those in other saline soils.

## Materials and methods

### Sampling and experimental design

**Fig. 1:**
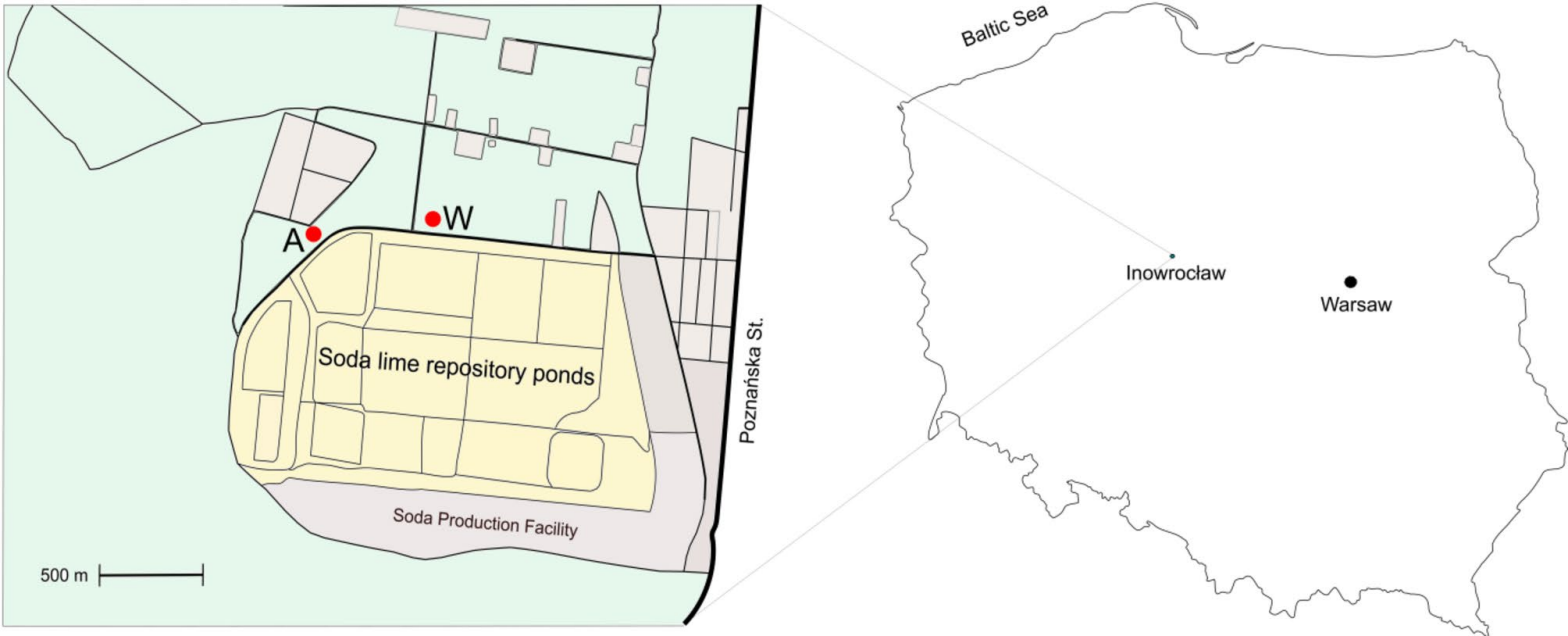
Outlook of the sampling sites. Figure 1. A - saline wasteland site with aster (*Tripolium pannonicum* Jacq.), W - arable field site with wheat (*Triticum aestivum* L.).

Aster (*Tripolium pannonicum* (Jacq.)), and wheat (*Triticum aestivum* L.) plants were collected in April 2023 from two distinct locations: a saline wasteland for Aster, and an arable field for wheat (Fig. 1).

The samples were obtained in Inowrocław, Poland, close to the soda lime repository ponds of the CIECH Soda company with four replicates for each species. Plants were placed in sterile polyethylene bags and stored in a portable travel refrigerator at 4 °C before laboratory analyses. The time from sampling to analysis did not exceed 90 min. Soil samples were collected from the root surface of each plant using a sterile spatula, and then transferred into sterile Eppendorf tubes for subsequent studies (four replicates per root system of the given species).

The experiment consisted of three stages: pretreatment, in which samples were enriched with bacterial cells; genomic DNA extraction; amplification and next-generation sequencing (NGS) analysis. Additionally, soil basic parameters, including ECe, pH, Corg, CaCO_3_, Ntot, and C/N were characterized.

### Soil characteristics

The soil samples for physicochemical analysis were dried and sieved through a 2.0 mm mesh screen and immediately stored at 4 °C for further analysis. Soil properties were analysed as described in Hulisz et al.^16^.

### Pretreatment, PVDF membrane preparation

Five grams of rhizosphere soil and four pieces of PVDF membrane were placed into 45 ml of sterile JMV medium in Simax bottles (100 mL) to enrich samples in nitrogen-fixing bacteria during the 24-hour incubation at 20 °C. The second set of bottles with 5 g of soil without any liquid served as a control.

The nitrogen-free JMV medium^17^ had the following composition (g L^−1^): mannitol, 5.0; K_2_HPO_4_, 0.6; KH_2_PO_4_, 1.8; MgSO_4_.7H_2_O, 0.2; NaCl, 10; CaCl_2_.2H_2_O, 0.02; micronutrient solution 2 mL (CuSO_4_.5H_2_O, 0.04; ZnSO_4_.7H_2_O, 0.12; H_3_BO_3_, 1.40; Na_2_MoO_4_.2H_2_O, 1.0; MnSO_4_.H_2_O, 1.175); bromothymol blue 2 mL (5 g L^−1^ in 0.2 N KOH); vitamin solution 1 mL (biotin, 0.1 gL^−1^). The pH was adjusted with KOH to 8.5 ± 0.2. The JMV medium was selected based on our prior experience, which demonstrated its effectiveness in the isolation of a diverse range of putative nitrogen-fixing bacteria from technogenic soil^18^. The JMV medium is highly selective for nitrogen-fixing bacteria due to several factors: a nitrogen-free environment, mannitol as a carbon source (which is favoured by nitrogen-fixing bacteria), an alkaline pH that supports exopolysaccharide production and stress resistance, essential micronutrients (especially molybdenum for nitrogenase activity), and salinity. This makes it particularly useful in saline or degraded soils, where other microbes may struggle due to osmotic stress, nutrient imbalances, or high pH^17,18^.

A polyvinylidene fluoride (PVDF) solution in dimethyl formamide (20% w/w) was prepared through slow dissolution by heating at 45 °C overnight. Based on previous studies, this concentration was selected as the optimal one for maximizing bacterial attachment^5^. The viscous polymeric solution was then cast onto a sheet of non-woven polyester fabric (Novatexx 2470, Freudenberg Filtration Technologies, Weinheim, Germany), taped to a glass plate, and evenly spread using a 100 μm casting roller (MeSep, Kraków, Poland). The membrane was immediately immersed in water and left to air-dry. Once dried, the membrane was cut into small pieces (~ 1 cm²), autoclaved, and prepared for use in the experiment.

### Genomic DNA extraction

A volume of 250 µl of the 10-fold soil suspension in JMV medium (control samples) or four pieces of PVDF membrane (membrane samples), retrieved from the JMV medium, were used for DNA extraction following the Qiagen protocol for soil samples (DNeasy PowerSoil Kit, Qiagen). These samples were designated as C_A or C_W (control aster and control wheat, respectively) and M_A or M_W (membrane aster and membrane wheat, respectively). The experiment was performed in four replicates for each of the two types of rhizosphere.

### NGS analysis

Metataxonomic analysis of nitrogen-fixing biota was evaluated by amplifying the *nif*H gene with the following primers: 5′ TGCGAYCCSAARGCBGACTC 3′ (PolF forward, without overhangs) and 5′ ATSGCCATCATYTCRCCGGA 3′ (PolR reverse, without overhangs) according to Poly et al.^19^.

The libraries were prepared following the Illumina Support Centre (ISC) protocol with a minor modification (2 × Phanta Max Master Mix, Vazyme Biotech, Nanjing City, China was applied instead of Kapa HifiHot Start Ready mix). Sequencing was performed by the Biobank (University of Łódź, Poland) on a MiSeq platform (Illumina, San Diego, CA, USA) using MiSeq Reagent Kit v2 (500 cycles) in paired-end mode (2 × 250 bp).

Data analysis of *nif*H gene amplicons with a yield of 1,626,305 raw reads was processed using the DADA2 R package^20^based on the DADA2 Pipeline Tutorial 1.16^21^. The filter and trim step was performed using the filterAndTrim command with maxN = 0, maxEE = c(2,2), trimLeft = c(20,20), truncQ = 2, rm.phix = TRUE, compress = TRUE, multithread = FALSE parameters. The nifH dada2 v2.05 database^22^ has been used as a reference dataset in the taxonomy assignment. Data was normalized using subsampling to 20,896 reads per sample.

### Statistical analyses

The rarefaction curves for the obtained reads, canonical correspondence analysis (CCA), principal coordinate analysis (PCoA), analysis of similarities (ANOSIM), and calculations of alpha diversity metrics, were done using vegan in R v. 4.1.0^23^. Differences in the α-diversity metrics between groups of samples were tested with analysis of variance (ANOVA) using Past v. 3.08^24^. In CCA the nitrifying biota at the ASV level was used as the dependent variable. The DNA extraction method and plant species were used as independent variables. The permutation test was applied to test the significance of constraints in the CCA model. Venn diagrams were generated in R using the limma package.

## Results

### Rhizosphere samples and sequencing efficiency

The most crucial physicochemical properties of the rhizosphere soil are presented in Table S1. This soil is classified as Mollic gleysol^16^, originating from alluvial sands and sandy loams. It is influenced by aeolian processes and the influx of technogenic materials rich in iron and CaCO_3_ from highly saline groundwater. The soil from the wasteland (aster rhizosphere) exhibited higher salinity (ECe 20.01 dS/m) compared to the soil from the arable field (wheat rhizosphere, ECe 0.86 dS/m). Both soils were characterized by a slightly alkaline pH (7.2–7.5), a high content of calcium carbonate (23.5 and 18.5 mg/g, respectively), and a similar C to N ratio (9–11). This ratio is typical for agricultural soils, indicating rapid organic matter mineralization.

Next-generation sequencing of amplicons from PCR products of the *nif*H gene resulted in 1,626,305 raw reads. Out of these, 424,033 reads were high-quality sequences. The mean read depth for the membrane samples was significantly higher at 65.6 Mbases (± 9.2 Mb) compared to the control samples at 51.0 Mbases (± 5.5 Mb) (t-test, t = 3.62, *p* = 0.005). The rarefaction curves of the *nif*H samples flattened at around 10,000 sequences in all types of samples, suggesting that high coverage of the sample’s diversity was captured (Fig.S1).

### DNA extraction method and plant species as potential drivers of community structure in rhizosphere soil

The results obtained from CCA revealed that, among the two factors that might have influenced the structure of nitrogen-fixing bacteria in the rhizosphere, plant species composition had a significant impact (Table 1).

**Table 1:** Significance of parameters used in this study, affecting bacterial community composition in rhizosphere soil samples, as revealed by CCA.

Target	Variable	Df	Chi^2^	F	Pr(> F)
*nif*H	DNA extraction method	1	0.225	0.839	0.686
	Plant species	1	0.987	3.685	0.001***
	Residual	11	2.947		

*p < 0.05; ** *p* < 0.01; ***p < 0.001.

The method of DNA extraction did not affect the composition of the nitrogen-fixing bacteria. This was supported by the PCoA analysis (Fig. S2) and the ANOSIM results. PCoA showed a clear separation of samples based on plant species but no separation based on the DNA extraction method. The ANOSIM statistic was R −0.2 (p-value 0.971) for aster and R −0.1 (p-value 0.6) for wheat.

**Fig. 2:**
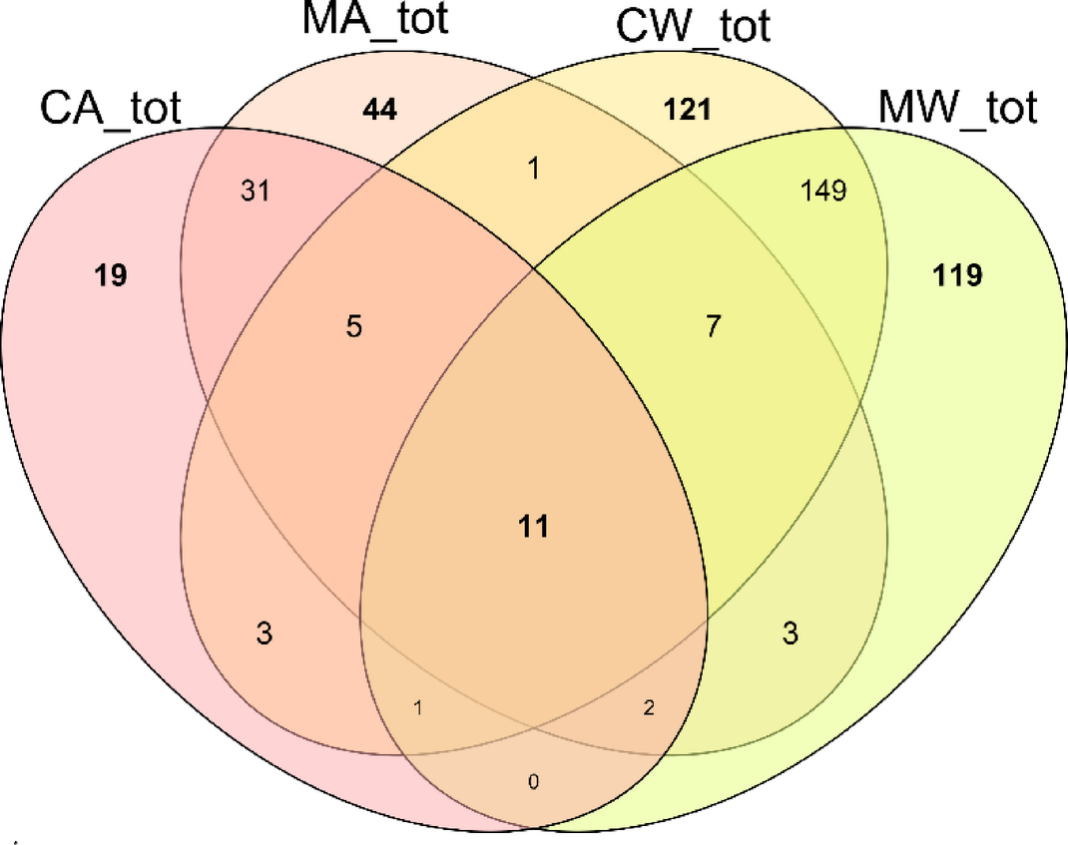
Venn diagrams presenting the number of unique elements (ASVs) in the rhizosphere of aster (A) and wheat (W) in control (C) and membrane (M) samples.

Venn diagrams (Fig. 2) unveiled distinctions in the number of amplicon sequence variants (ASVs) between control and PVDF membrane treatment for *nif*H gene amplicons.

The median value of the unique elements in aster membrane samples was significantly higher than in control samples (22 vs. 11; Mann-Whitney U = 98.5, *p* = 0.026). The number of unique elements in wheat rhizosphere samples did not differ between membrane samples and controls (mean value 84 vs. 81; t-test: t = 0.12, *p* = 0.903). Unique ASVs in aster and wheat membrane samples constituted 8.5% and 23%, respectively, of all ASVs in the dataset. Further analysis showed that there were no significant differences in diversity metrics in soil rhizosphere samples between the methods of DNA extraction (Table 2).

**Table 2:** Alpha diversity metrics in rhizosphere soil samples.

Target			Sobs	Shannon-Wienner H’	Simpson 1-D
*nif*H	Aster	C	30 ± 16	1.8 ± 0.6	0.730 ± 0.175
		M	44 ± 27	1.9 ± 0.5	0.747 ± 0.162
	Wheat	C	143 ± 60	2.8 ±0.7	0.830 ± 0.093
		M	144 ± 47	2.9 ± 0.9	0.785 ± 0.192

Differences between control and membrane samples were not significant (p >0.05).

### Structure of bacterial communities – nitrogen-fixing bacteria

**Fig. 3:**
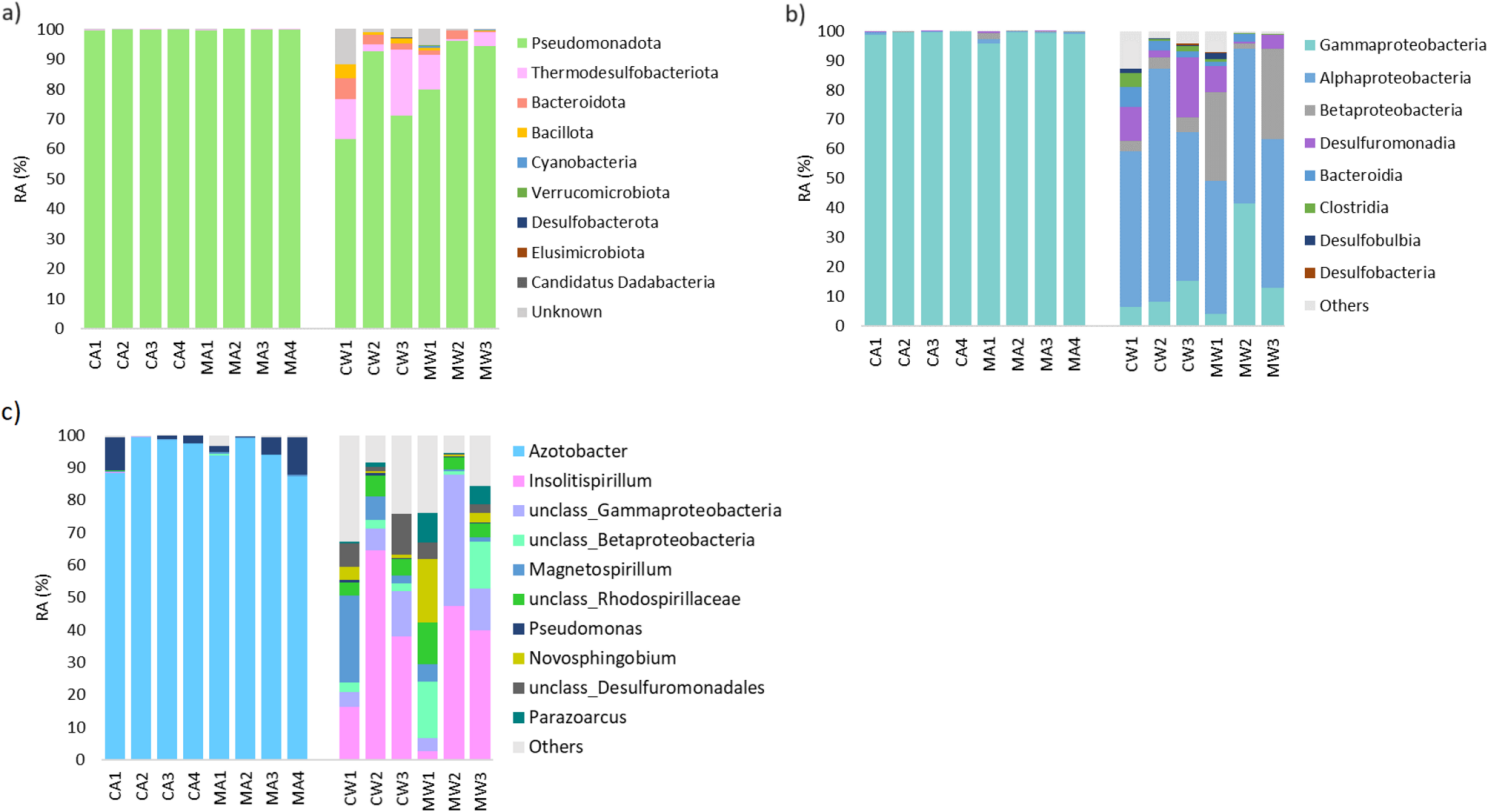
Structure of the putative nitrogen-fixing bacterial communities at the phylum (**a**), class (**b**) and genus (**c**) levels in the rhizosphere of aster (A) and wheat (W) in control (C) and membrane (M) samples.

At the phylum level, nitrogen-fixing bacteria were dominated by reads assigned to Pseudomonadota (Fig. 3; Table S2). The Aster rhizosphere exhibited a nearly complete dominance of this phylum compared to the wheat rhizosphere (median 99.91% versus 86.11%). Representatives of the phyla Thermodesulfobacteriota and Bacillota were present in both types of rhizosphere; however, they were more abundant in the wheat rhizosphere compared to the aster (median 8.20% versus 0.08%, respectively, and 0.87% versus 0.00%). Phylotypes assigned to Bacteroidota, Cyanobacteria, Verrucomicrobiota, Desulfobacterota, and Elusimicrobiota were exclusively present in the wheat rhizosphere.

In terms of class-level taxonomy, the majority of reads in aster samples belonged to Gammaproteobacteria (median value of 99.48%). In wheat rhizosphere samples, Alphaproteobacteria was the most abundant class (51.48%), with Desulfuromonadia ranking second (6.65%). Representatives of other top ten bacterial classes, namely Alphaproteobacteria, Betaproteobacteria, and Desulfuromonadia, were more abundant in the wheat rhizosphere compared to the aster rhizosphere. Phylotypes assigned to Bacteroidia, Clostridia, Desulfobulbia, and Desulfobacteria were exclusively detected in the wheat rhizosphere.

The rhizosphere of Aster was predominantly occupied by *Azotobacter* reads, accounting for a median value of 95.69%. The second most abundant genus was *Pseudomonas*, constituting 2.02% of the community. In the wheat rhizosphere, the most prevalent genus was *Insolitispirillum* with 38.80%, followed by unclassified ASVs within the class Gammaproteobacteria (9.76%) and the family *Rhodospirillaceae* (4.74%), respectively.

The rare, putative nitrogen-fixing bacteria, constituting less than 0.01% of the relative abundance in a given sample and found exclusively in the membrane samples, are presented in Table 3.

**Table 3:** Rare (< 0.01% RA) nitrogen-fixing bacteria exclusively present in membrane samples of aster and wheat in Inowrocław technosoil.

Target	ASV	Taxonomy
Aster	ASV402	*Parazoarcus*
	ASV455	unclassified *Hydrogenophilales*
	ASV467	*Geobacter*
	ASV487	unclassified *Geminicoccaceae*
	ASV522	*Caenispirillum*
	ASV143	*Halorhodospira*
	ASV366	*Parazoarcus*
	ASV469	unclassified *Anaerolineae*
Wheat	ASV435	*Leptolyngbya*
	ASV461	*Citrifermentans*
	ASV470	*Anabaena*
	ASV474	*Geobacter*
	ASV477	unclassified *Desulfuromonadaceae*
	ASV493	unclassified *Chromatiales*
	ASV496	*Geobacter*
	ASV524	unclassified Elusimicrobiota
	ASV525	unclassified *Terasakiellaceae*
	ASV411	*Methyloferula*
	ASV441	*Candidatus ‘*Dadabacteria’
	ASV491	*Methylocystis*
	ASV492	*Desulfovibrio*
	ASV513	*Desulfopila*
	ASV515	*Desulfovibrio*
	ASV516	*Geobacter*
	ASV517	*Geobacter*

## Discussion

Assessing nitrogen-fixing microbial community composition through amplicon sequencing of the nifH gene is a widely used technique in microbial ecology. However, several inherent challenges and limitations can affect the accuracy and reliability of the results. The most important encompass primer bias, PCR amplification bias, and sequencing errors^25,26^.

Rare species are often present in very low abundances (< 0.01% RA), making their detection highly dependent on the sequencing depth. Insufficient sequencing depth may fail to capture these low-abundance taxa, leading to an incomplete picture of the community diversity^27^. During PCR amplification, rare species may be disproportionately underrepresented compared to more abundant taxa. This is due to the stochastic nature of PCR, where low-abundance sequences are less likely to be amplified consistently across cycles, especially when starting from a small number of template molecules^28^. The results of the sequencing of the *nif*H gene appeared successful in capturing the diversity of the putative nitrogen fixers. This indicates that the sequencing approach was well-designed and performed reliably. The method of DNA isolation, as indicated by CCA, PCoA, ANOSIM, and analysis of alpha diversity metrics, did not significantly affect the overall community composition.

However, Venn diagrams revealed notable differences in the number of bacterial ASVs uniquely detected in samples where metagenomic DNA was isolated using a membrane. In wheat membrane samples, unique ASVs accounted for 23% of all ASVs in the dataset, suggesting that the PVDF membrane method enabled the detection of a substantial portion of the bacterial community not captured by the standard method. Similarly, in aster membrane samples, unique ASVs constituted 8.5% of all ASVs. Although less pronounced than in wheat samples, this still highlights the membrane method’s ability to uncover additional bacterial diversity compared to conventional approaches.

Alpha diversity metrics, such as the Shannon and Simpson indices, evaluate both the richness and evenness of taxa^29^. If the unique ASVs detected in membrane samples are primarily rare and of low abundance, their contribution to these metrics may be minimal. In contrast, control samples with a more even distribution of taxa, despite having fewer unique ASVs, could exhibit similar or even higher alpha diversity values. In this study, both membrane and control samples were dominated by a few highly abundant taxa, which likely constrained the ability of alpha diversity metrics to capture differences arising from rare, unique ASVs. Consequently, the additional diversity detected in membrane samples may have been masked by the dominance of a few taxa. Furthermore, both CCA and PCoA analyses are heavily influenced by the most abundant taxa, as these methods aim to explain the largest variation in the dataset^30^. Rare taxa, which contribute minimally to overall variation, have a limited impact on ordination results. This limitation is common in community ecology, as rare taxa typically represent a small fraction of the data matrix and do not significantly influence the patterns highlighted by ordination methods.

It is well established that nonspecific interactions or without the involvement of any particular stereospecific macromolecular binding sites mediate the attachment between membrane and bacteria. Therefore, the attachment is not substrate-specific which leads to the easy attachment of various kinds of bacteria on the membrane surface^31^. The deposition and accumulation of microorganisms onto the membrane surface involves a series of mechanisms causing irreversible attachment of the bacteria at the surface and inside the pores of the membrane^32^. Secondly, the amount of samples collected for any microbial studies comprises a very small amount which might not represent all types of bacteria^5^. Therefore, the membrane helps to concentrate/enrich different types of bacteria from the surrounding sample. Subsequently, it results in the isolation of bacteria representing diverse groups. Also, among several polymeric membranes, PVDF attached more diverse bacteria according to several studies conducted previously^5,33^. In those specific studies, the PVDF (20% W/W) membrane effectively and successfully isolated diversified bacteria from seawater samples. However, the studies were based on biochemical methods and identification (16 S rRNA) of bacteria recovered from the membrane compared to the surrounding water samples. The present study was an approach to validate those findings with the help of molecular-based studies and whether this applies to soil samples too. Interestingly, the results of the present study accompany the fact that membranes especially PVDF (20% W/W) can concentrate diverse bacteria from the soil samples.

Plants profoundly influence the soil microbiome through root exudates, which are complex mixtures of sugars, amino acids, carboxylic acids, and secondary metabolites. These compounds serve as signalling molecules, attractants, stimulants, or inhibitors that shape the microbial community structure. The rhizobiome, or the microbial community in the rhizosphere, is shaped by plant-specific exudates, whose composition is under genetic control and varies across plant species^34,35^. Additionally, the rhizobiome is influenced by soil type and geographical distance^36^.

Aster, a halophytic plant, thrives in natural and anthropogenic environments near saline water sources, such as coastal areas or soda factory waste ponds^37,38^. Halophytes like aster possess salinity-responsive genes and proteins that enable survival in high-salt environments. Their rhizospheres host plant growth-promoting rhizobacteria (PGPR) which enhance salinity tolerance and mitigate environmental stress^39^.

In saline technogenic soils, the nitrogen-fixing bacterial community in the aster rhizosphere was characterized by a predominance of halotolerant bacteria, including members of the *Azotobacter *genus. These bacteria exhibit adaptations such as increased energy capacity to withstand saline conditions, which otherwise suppress microbial biomass and nitrogenase activity^40,41^. Previous studies from the same area identified a halotolerant strain, *Azotobacter chroococcum*W4ii, with plant growth-promoting properties, suggesting a vital role for such bacteria in supporting aster growth in saline technogenic soils^18^. Rare putative nitrogen-fixing bacteria, including *Parazoarcus*^42^, *Geobacter*^43^, and *Halorhodospira*^44^, were identified using the membrane-based approach. Despite their low abundance, these bacteria appear to be key members of the aster rhizosphere community, given their documented roles in nitrogen fixation and adaptation to saline environments.

Wheat, a crop with moderate sensitivity to salinity, experiences significant reductions in growth and yield under saline conditions. This sensitivity arises from ionic and osmotic stress that disrupts water uptake and nutrient balance^45^. Various strategies, including salt-tolerant endophytes and genetic modifications, have been employed to enhance wheat’s salinity tolerance^46,47^. Wheat requires a balanced supply of nutrients, particularly nitrogen, for optimal growth. Over-application of nitrogen fertilizers, a common practice in wheat cultivation, alters soil microbial activity and ecological functions^48^. This dynamic impacts the rhizosphere microbiome, with wheat root exudates potentially attracting diverse nitrogen-fixing bacteria to compensate for nitrogen deficiencies^49^.

The nitrogen-fixing bacterial communities in the rhizospheres of aster and wheat differed significantly. The aster rhizosphere, dominated by halotolerant *Azotobacter* and *Pseudomonas *species, reflected its adaptation to high-salinity environments. In contrast, wheat exhibited a broader spectrum of nitrogen-fixing bacteria, likely influenced by its nutrient demands and sensitivity to saline stress. This distinction underscores the role of root exudation in shaping rhizosphere composition, though further research is needed to elucidate how domestication and metabolic specialization drive these interactions^50^. Among the nitrogen-fixing bacteria in wheat rhizosphere soil, *Insolitispirillum *emerged as a dominant genus. Though relatively understudied^51^, its prevalence suggests a significant ecological role within the *Rhodospirillaceae *family, which includes many known nitrogen-fixing species^52^. Rare taxa in wheat rhizosphere revealed with the improved detection method assigned to the genera *Anabaena* and *Leptolyngbya *are both well-known diazotrophic cyanobacteria^47^ that convert atmospheric nitrogen into ammonium, making it available for plant uptake. Their ability to form biofilms may also help to retain moisture and nutrients in saline soils. Additionally, *Methyloferula *and *Methylocystis*, methane-oxidizing bacteria capable of nitrogen fixation^48^, were identified. These bacteria may play a dual role in the rhizosphere by contributing to both nitrogen and carbon cycling, further supporting soil health and plant growth. At the broader taxonomic level, Gammaproteobacteria and Alphaproteobacteria were the dominant diazotrophic bacteria in coastal saline soils^47^, a trend mirrored in the rhizospheres of both plants. However, variations in salinity levels significantly influenced community structure. For instance, higher salinity in our samples (approximately three times higher than coastal saline soils) likely shifted diazotrophic bacterial dominance, as observed in the relative abundance of Betaproteobacteria and *Burkholderia *in Polish arable soils^48^.

## Conclusions

The findings of this study confirm that PVDF membranes are an effective tool for gaining insights into nitrogen-fixing communities and rare taxa. The interaction between plants and their rhizosphere microbiomes is highly dynamic, shaped by root exudates, soil conditions, and environmental stressors. In saline soils, aster supports a specialized, halotolerant nitrogen-fixing community, while wheat relies on a more diverse set of nitrogen-fixing bacteria to meet its nutrient needs.

The presence of rare nitrogen fixers, including cyanobacteria and methane-oxidizing bacteria, highlights the complex ecological interactions that sustain plant health and soil fertility. Further research is needed to understand how plant-specific factors, domestication, and environmental pressures influence the assembly of these microbial communities. Future studies should also focus on optimizing sequencing and analytical approaches to better integrate the contributions of rare taxa into broader ecological analyses.

PVDF membranes offer significant advantages for microbial diversity studies, particularly in challenging environments. Their unique properties, such as high chemical resistance and durability, may enable efficient microbial cell capture in extreme conditions, including highly saline and alkaline soils.

## Electronic supplementary material

Below is the link to the electronic supplementary material.

Supplementary Material 1

## Data Availability

Sequencing data were deposited in NCBI under accession number PRJNA1049880 (https://www.ncbi.nlm.nih.gov/bioproject/PRJNA1049880/).

## References

[1] Ji, J., Liu, F., Hashim, N. A., Abed, M. M. & Li, K. Poly (vinylidene fluoride) (PVDF) membranes for fluid separation. *React. Funct. Polym.* **86**, 134–153 (2015).

[2] Ruan, L. et al. Properties and applications of the Β phase poly(vinylidene fluoride). *Polymers* **10**, 228 (2018). 30966263 10.3390/polym10030228 PMC6415445

[3] Dallaev, R. et al. Brief review of PVDF properties and applications potential. *Polymers* **14** (22), 4793 (2022). 36432920 10.3390/polym14224793 PMC9698228

[4] Xiang, S. et al. Fabrication of PVDF/EVOH blend Hollow fiber membranes with hydrophilic property via thermally induced phase process. *Sep. Purif. Technol.* **301**, 122031 (2022).

[5] Kumar, S. B., Sharnagat, P., Manna, P., Bhattacharya, A. & Haldar, S. Enhanced bacterial affinity of PVDF membrane: its application as improved sea water sampling tool for environmental monitoring. *Environ. Sci. Pollut Res. Int.* **24** (6), 5831–5840 (2017). 28054272 10.1007/s11356-016-8318-1

[6] Zhao, G. & Chen, W. N. Biofouling formation and structure on original and modified PVDF membranes: role of microbial species and membrane properties. *RSC Adv.* **7**, 37990–38000 (2017).

[7] Krasowska, A. & Sigler, K. How microorganisms use hydrophobicity and what does this mean for human needs? *Front. Cell. Infect. Microbiol.* **4**, 112 (2014). 25191645 10.3389/fcimb.2014.00112 PMC4137226

[8] AlSawaftah, N., Abuwatfa, W., Darwish, N. & Husseini, G. A. A review on membrane biofouling: prediction, characterization, and mitigation. *Membr* **12** (12), 1271 (2022). 10.3390/membranes12121271 PMC9787789 36557178

[9] Kumar, S. B., Shinde, A. H., Mehta, R., Bhattacharya, A. & Haldar, S. Simple, one-step dye-based kit for bacterial contamination detection in a range of water sources. *Sens. Actuators Chem.* **276**, 121–127 (2018).

[10] Kumar, S. B., Shinde, A. H., Behere, M. J., Italia, D. & Haldar, S. Simple, rapid and on spot dye-based sensor for the detection of *Vibrio* load in shrimp culture farms. *Arch. Microbiol.* 203(6), 3525–3532 (2021). (2021). 10.1007/s00203-021-02333-3 33942157

[11] Wydro, U. & Soil Microbiome Study Based on DNA Extraction. *Rev. Water*, **14**(24), 3999 (2022).

[12] 12, Kuan, K. B., Othman, R., Abdul Rahim, K. & Shamsuddin, Z. H. Plant growth-promoting rhizobacteria inoculation to enhance vegetative growth, nitrogen fixation and nitrogen remobilisation of maize under greenhouse conditions. *PLoS One* **24**,11(3), e0152478 (2016). 10.1371/journal.pone.0152478 PMC4807084 27011317

[13] Singh, R. K. et al. Diversity of nitrogen-fixing rhizobacteria associated with sugarcane: a comprehensive study of plant-microbe interactions for growth enhancement in *Saccharum* spp. *BMC Plant. Biol.* **20**, 1–21 (2020). 32423383 10.1186/s12870-020-02400-9 PMC7236179

[14] Shi, Z. et al. Screening of high-efficiency nitrogen-fixing bacteria from the traditional Chinese medicine plant *Astragalus mongolicus* and its effect on plant growth promotion and bacterial communities in the rhizosphere. *BMC Microbiol.* **23** (1), 292 (2023). 37845638 10.1186/s12866-023-03026-1 PMC10578054

[15] Kalwasińska, A. et al. Technogenic soil salinisation, vegetation, and management shape microbial abundance, diversity, and activity. *Sci. Total Environ.* **905**, 167380 (2023). 37774878 10.1016/j.scitotenv.2023.167380

[16] Hulisz, P., Pindral, S., Kobierski, M. & Charzynski, P. Technogenic layers in organic soils as a result of the impact of the soda industry. *Eurasian Soil. Sci.* **51**, 1133–1141 (2018).

[17] Baldani, J. I., Reis, V. M., Videira, S. S., Boddey, L. H. & Baldani, V. L. D. The Art of isolating nitrogen-fixing bacteria from non-leguminous plants using N-free semi-solid media: a practical guide for microbiologists. *Plant. Soil.* **384**, 413–431 (2014).

[18] Binod Kumar, S., Kalwasińska, A., Brzezinska, M. S. & Wróbel, M. Application of halotolerant *Azotobacter chroococcum* W4ii isolated from technosoils to mitigate salt stress in wheat plant. *Open Res. Eur*. 3(76), 76 (2023). (2023). 10.12688/openreseurope.15821.4 PMC11325138 39148935

[19] Poly, F., Monrozier, L. J. & Bally, R. Improvement in the RFLP procedure for studying the diversity of NifH genes in communities of nitrogen fixers in soil. *Res. Microbiol.* **152** (1), 95–103 (2001). 11281330 10.1016/s0923-2508(00)01172-4

[20] Callahan, B. J. et al. DADA2: High-resolution sample inference from illumina amplicon data. *Nat. Methods*. **13** (7), 581–583 (2016). 27214047 10.1038/nmeth.3869 PMC4927377

[21] DADA2 Pipeline Tutorial 1.16. https://benjjneb.github.io/dada2/tutorial.html

[22] Moynihan, M. A. & Reeder, C. F. moyn413/nifHdada2: v2.0.5. Zenodo, (2023). https://zenodo.org/records/7996213

[23] Oksanen, J. et al. Vegan: Community Ecology Package. R package version 2.5-6 (2019). https://github.com/vegandevs/vegan

[24] Hammer, Ø. Paleontological statistics software package for education and data analysis. *Palaeontol. Electron.* **4**, 9 (2001).

[25] Poretsky, R., Rodriguez-R, L. M., Luo, C., Tsementzi, D. & Konstantinidis, K. T. Strengths and limitations of 16S rRNA gene amplicon sequencing in revealing Temporal microbial community dynamics. *PloS One* **9**(4), e93827 (2014). 10.1371/journal.pone.0093827 PMC3979728 24714158

[26] Smith, D. P. & Peay, K. G. Sequence depth, not PCR replication, improves ecological inference from next generation DNA sequencing. *PloS One* **9**(2), e90234 (2014). 10.1371/journal.pone.0090234 PMC3938664 24587293

[27] Pinto, A. J. & Raskin, L. PCR biases distort bacterial and archaeal community structure in pyrosequencing datasets. *PLoS One*. **7** (8), e43093 (2012). 22905208 10.1371/journal.pone.0043093 PMC3419673

[28] Chiu, C. H. & Chao, A. Estimating and comparing microbial diversity in the presence of sequencing errors. *PeerJ* **1**, 4:e1634 (2016). 10.7717/peerj.1634 PMC4741086 26855872

[29] Verniest, F. & Greulich, S. Methods for assessing the effects of environmental parameters on biological communities in long-term ecological studies - A literature review. *Ecol. Model.* **414**, 108732 (2019).

[30] Mallevialle, J., Odendaal, P. E. & Wiesner, M. R. *Water Treatment Membrane Processes* (McGraw-Hill, 1996).

[31] Rosales, A. B. et al. Minimizing bacterial adhesion on membrane: multiscale characterization of surface modifications. *J. Membr. Sci.* **684**, 121867 (2023).

[32] Kumar, S. B., Shinde, A. H., Mehta, R., Bhattacharya, A. & Haldar, S. Simple, one-step dye-based kit for bacterial contamination detection in a range of water sources. *Sens. Actuators B Chem.* **276**, 121–127 (2028).

[32] Kumar, S. B., Trivedi, H., Baraiya, N. R. & Haldar, S. An improved device with an affinity membrane to collect depth specific contamination free water for environmental assessment. *Analyst* 143(3), 662–669 (2018). (2018). 10.1039/c7an01528c 29309074

[34] Hu, L. et al. Root exudate metabolites drive plant-soil feedbacks on growth and defense by shaping the rhizosphere microbiota. *Nat. Commun.* **9** (1), 2738 (2018). 30013066 10.1038/s41467-018-05122-7 PMC6048113

[35] Zhao, X. et al. Introduction of *Panax notoginseng* into pine forests significantly enhances the diversity, stochastic processes, and network complexity of nitrogen-fixing bacteria in the soil. *Front Microbiol*. ;16:1531875 (2025). (2025). 10.3389/fmicb.2025.1531875 PMC11830724 39963494

[36] Qin, K., Dong, X., Jifon, J. & Leskovar, D. I. Rhizosphere microbial biomass is affected by soil type, organic and water inputs in a bell pepper system. *Appl. Soil. Ecol.* **138**, 80–87 (2019).

[37] Lazarus, M. & Wszałek-Rożek, K. Two rare halophyte species: Aster tripolium L. and Plantago maritima L. on the Baltic Coast in Poland–their resources, distribution and implications for conservation management. *Biodiv Res. Conserv.* **41(1)**, 51–60 (2016). (2016).

[38] Karasińska, W., Nienartowicz, A., Kunz, M., Kamiński, D. & Piernik, A. Resources and dynamics of halophytes in agricultural and industrial landscapes of the Western part of Kujawy, central Poland. *Ecol. Quest*. **32** (4), 7–25 (2021).

[39] Etesami, H. & Glick, B. R. Halotolerant plant growth–promoting bacteria: Prospects for alleviating salinity stress in plants. *Environ. Exp. Bot.* 178, 104124 (2020). (2020).

[40] Severin, I., Confurius-Guns, V. & Stal, L. J. Effect of salinity on nitrogenase activity and composition of the active diazotrophic community in intertidal microbial Mats. *Arch. Microbiol.* **194** (6), 483–491 (2012). 22228487 10.1007/s00203-011-0787-5 PMC3354318

[41] Rath, K. M., Maheshwari, A., Bengtson, P. & Rousk, J. Comparative toxicities of salts on microbial processes in soil. *Appl. Environ. Microbiol.* **82** (7), 2012–2020 (2016). 26801570 10.1128/AEM.04052-15 PMC4807522

[42] Boden, R., Hutt, L. P. & Rae, A. W. Reclassification of *Thiobacillus aquaesulis* (Wood & Kelly, 1995) as *Annwoodia aquaesulis* gen. Nov., comb. Nov., transfer of *Thiobacillus* (Beijerinck, 1904) from the *Hydrogenophilales* to the Nitrosomonadales, proposal of *Hydrogenophilalia* class. Nov. Within the ‘proteobacteria’, and four new families Within the orders *Nitrosomonadales* and *Rhodocyclales*. *Int. J. Syst. Evol. Microbiol.* **67** (5), 1191–1205 (2017). 28581923 10.1099/ijsem.0.001927

[43] Ortiz-Medina, J. F., Poole, M. R., Grunden, A. M. & Call, D. F. Nitrogen fixation and ammonium assimilation pathway expression of *Geobacter sulfurreducens* changes in response to the anode potential in microbial electrochemical cells. *Appl. Environ. Microbiol.* **26** (4), e0207322 (2023). 10.1128/aem.02073-22 PMC10132095 36975810

[44] Tsuihiji, H., Yamazaki, Y., Kamikubo, H., Imamoto, Y. & Kataoka, M. Cloning and characterization of nif structural and regulatory genes in the purple sulfur bacterium, *Halorhodospira halophila*. *J. Biosci. Bioeng.* ; 101, 263–270 (2006). (2006). 10.1263/jbb.101.263 16716929

[45] Duarte-Delgado, D. et al. Transcriptome profiling at osmotic and ionic phases of salt stress response in bread wheat uncovers trait-specific candidate genes. *BMC Plant. Biol.* **16** (20(1)), 428 (2020). (2020). 10.1186/s12870-020-02616-9 PMC7493341 32938380

[46] Bouzouina, M., Kouadria, R. & Lotmani, B. Fungal endophytes alleviate salt stress in wheat in terms of growth, ion homeostasis and osmoregulation. *J. Appl. Microbiol.* **130** (3), 913–925 (2021). 32743928 10.1111/jam.14804

[47] Yu, Y., Wu, Y. & He, L. A wheat WRKY transcription factor TaWRKY17 enhances tolerance to salt stress in Transgenic *Arabidopsis* and wheat plant. *Plant. Mol. Biol.* **113** (4–5), 171–191 (2023). 37902906 10.1007/s11103-023-01381-1

[48] Zhou, J. et al. Consistent effects of nitrogen fertilization on soil bacterial communities in black soils for two crop seasons in China. *Sci. Rep.* **7** (1), 3267 (2017). 28607352 10.1038/s41598-017-03539-6 PMC5468298

[49] Yang, Y. et al. Effect of nitrogen management on wheat yield, water and nitrogen utilization, and economic benefits under ridge-furrow cropping system with supplementary irrigation. *Agronomy* **13** (7), 1708 (2023).

[50] Yue, H. et al. Plant domestication shapes rhizosphere Microbiome assembly and metabolic functions. *Microbiome* **11** (1), 70 (2023). 37004105 10.1186/s40168-023-01513-1 PMC10064753

[51] Koziaeva, V. V., Sorokin, D. Y., Kolganova, T. V. & Grouzdev, D. S. *Magnetospirillum sulfuroxidans* Sp. nov., capable of sulfur-dependent lithoautotrophy and a taxonomic Reevaluation of the order *Rhodospirillales*. *Syst. Appl. Microbiol.* **46** (3), 126406 (2023). 36898262 10.1016/j.syapm.2023.126406

[52] Madigan, M., Cox, S. S. & Stegeman, R. A. Nitrogen fixation and nitrogenase activities in members of the family. *Rhodospirillaceae J. Bacteriol.* **157**, 73–78 (1984). 6581158 10.1128/jb.157.1.73-78.1984 PMC215131

[53] Charpy, L. et al. Dinitrogen-fixing cyanobacteria in microbial Mats of two shallow coral reef ecosystems. *Microb. Ecol.* **59** (1), 174–186 (2010). 19705191 10.1007/s00248-009-9576-y PMC2807599

[54] Mäkipää, R. et al. Methanotrophs are core members of the Diazotroph community in decaying Norway Spruce logs. *Soil. Biol. Biochem.* **120**, 230–232 (2018).

[55] Song, Y. et al. The diversity and structure of diazotrophic communities in the rhizosphere of coastal saline plants is mainly affected by soil physicochemical factors but not host plant species. *Front. Mar. Sci.* **9**, 1100289 (2022).

[56] Wolińska, A. et al. Metagenomic analysis of some potential nitrogen-fixing bacteria in arable soils at different formation processes. *Microb. Ecol.* **73**, 162–176 (2017). 27581036 10.1007/s00248-016-0837-2 PMC5209426

