# Supplementary material for: Enhanced understanding of nitrogen fixing bacteria through DNA extraction with polyvinylidene fluoride membrane
Source: Sci Rep. 2025 May 8;15:16079. doi: 10.1038/s41598-025-00173-5 (PMC12062204; doi:10.1038/s41598-025-00173-5)
Supplement: Supplementary file 1 — Supplementary Material 1 [file 41598_2025_173_MOESM1_ESM.docx]

**Supplementary Materials**

**Enhanced Understanding of Nitrogen Fixing Bacteria Through DNA Extraction with Polyvinylidene Fluoride Membrane**

**Agnieszka Kalwasińska^a*^, Igor Królikiewicz^a^, Sushma Rani Tirkey^a^ Attila Szabó**^b,c^**, Sweta Binod Kumar^a^**

^a^Department of Environmental Microbiology and Biotechnology, Faculty of Biological and Veterinary Sciences, Nicolaus Copernicus University in Toruń, Lwowska 1, 87-100 Toruń, Poland

^b^Department of Aquatic Sciences and Assessment, Swedish University of Agricultural Sciences, 750 07 Uppsala, Sweden

^c^Institute of Aquatic Ecology, Centre for Ecological Research, Karolina út 29, 1113 Budapest, Hungary

*Corresponding author: Agnieszka Kalwasińska, [kala@umk.pl](mailto:kala@umk.pl), +48566112521


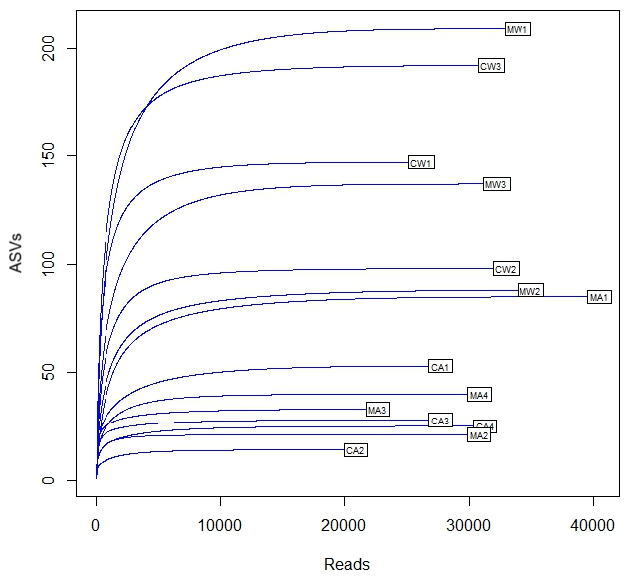


Fig. S1. Rarefaction curves of the *nif*H gene amplicon sequencing


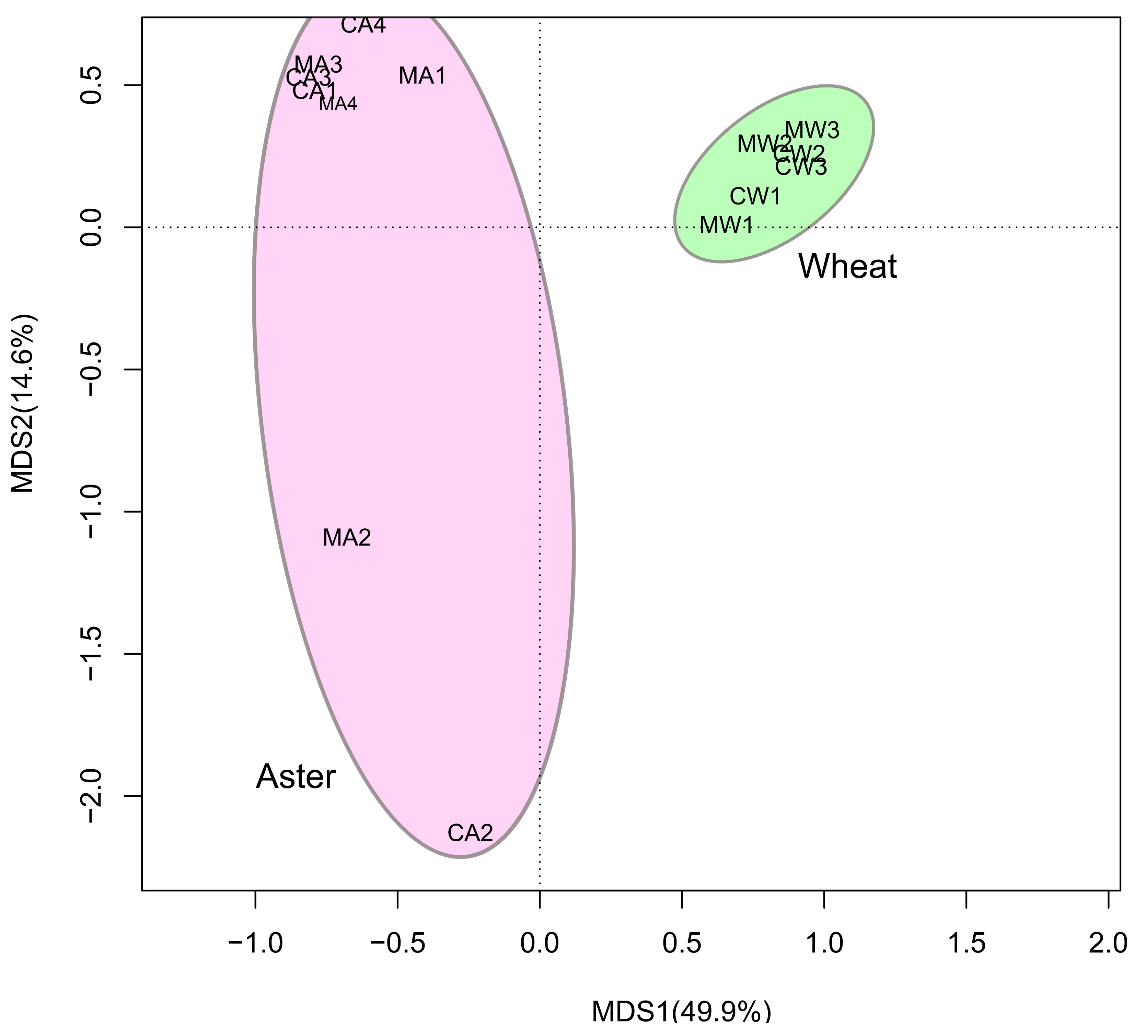


Fig. S2. PCoA ordination diagram based on the Bray-Curtis distance

Table S1. Soil characteristics

| Classification (WRB) | Mollic gleysol | | | |  |
| --- | --- | --- | --- | --- | --- |
| Location | |  | Wasteland | Arable field | |
| ECe | | dS/m | 20.01 | 0.86 | |
| pH (H_2_O 1:5) | |  | 7.24 | 7.49 | |
| C org | | mg/g | 9.93 | 11.35 | |
| CaCO_3_ | | mg/g | 23.5 | 18.45 | |
| N tot | | mg/g | 0.94 | 1.25 | |
| C/N | | mg/g | 11.6 | 9.00 | |

Table S2. Top10 putative nitrogen fixing phyla, classes, and genera in the rhizosphere soil of aster and wheat in technosoil from Inowrocław

| Taxon | Aster | Wheat | U | p-value |  |
| --- | --- | --- | --- | --- | --- |
| Pseudomonadota | 99.91 | 86.11 | 48 | 0.002 | ** |
| Thermodesulfobacteriota | 0.08 | 8.20 | 0 | 0.002 | ** |
| Unknown | 0.01 | 1.95 | 0 | 0.002 | ** |
| Bacteroidota | 0.00 | 2.26 | 0 | 0.008 | ** |
| Bacillota | 0.00 | 0.87 | 0 | 0.002 | ** |
| Cyanobacteria | 0.00 | 0.00 | 16 | 0.112 |  |
| Verrucomicrobiota | 0.00 | 0.00 | 16 | 0.112 |  |
| Desulfobacterota | 0.00 | 0.01 | 12 | 0.039 |  |
| Elusimicrobiota | 0.00 | 0.00 | 16 | 0.112 |  |
| Candidatus Dadabacteria | 0.00 | 0.00 | 20 | 0.312 |  |
|  |  |  |  |  |  |
| Gammaproteobacteria | 99.48 | 10.57 | 48 | 0.002 | ** |
| Alphaproteobacteria | 0.14 | 51.48 | 0 | 0.002 | ** |
| Betaproteobacteria | 0.27 | 4.49 | 1 | 0.004 | ** |
| Desulfuromonadia | 0.08 | 6.65 | 0 | 0.002 | ** |
| Bacteroidia | 0.00 | 2.26 | 0 | 0.001 | ** |
| Clostridia | 0.00 | 0.86 | 0 | 0.001 | ** |
| Desulfobulbia | 0.00 | 0.23 | 0 | 0.001 | ** |
| Desulfobacteria | 0.00 | 0.12 | 4 | 0.003 | ** |
|  |  |  |  |  |  |
| *Azotobacter* | 95.69 | 0.12 | 48 | 0.002 | ** |
| *Insolitispirillum* | 0.00 | 38.80 | 0 | 0.002 | ** |
| unclass_Gammaproteobacteria | 0.00 | 9.76 | 0 | 0.001 | ** |
| unclass_Betaproteobacteria | 0.01 | 2.77 | 0 | 0.002 | ** |
| *Magnetospirillum* | 0.14 | 3.83 | 2 | 0.005 | ** |
| *unclass_Rhodospirillaceae* | 0.00 | 4.74 | 0 | 0.001 | ** |
| *Pseudomonas* | 2.02 | 0.32 | 40 | 0.038 | * |
| *Novosphingobium* | 0.00 | 2.13 | 0 | 0.001 | ** |
| unclass_*Desulfuromonadales* | 0.00 | 3.88 | 0 | 0.002 | ** |
| *Parazoarcus* | 0.00 | 0.89 | 0 | 0.001 | ** |

Significant differences between median values of relative abundance in the rhizosphere of aster and wheat were indicated with asterisks *p<0.05; ** p<0.01; ***p<0.001
